# Functional Nausea Is Real and Makes You Sick

**DOI:** 10.3389/fped.2022.848659

**Published:** 2022-02-25

**Authors:** Carlo Di Lorenzo

**Affiliations:** Division of Gastroenterology, Hepatology, and Nutrition, Department of Pediatrics, Nationwide Children's Hospital, Columbus, OH, United States

**Keywords:** nausea, functional - child, dyspepsia, pediatrics - children, gastroparesis

## Abstract

Functional nausea is a condition that severely impairs the quality of life of affected individuals. Only recently, it has been added to the pediatric list of disorders of gut-brain interaction. In most cases, only minimal testing is needed to diagnose functional nausea. Hypnotherapy has been shown to be a very effective treatment and there are several other medical and non-medical interventions which have the potential to benefit sub-groups of patients with chronic nausea.

## Introduction

There are very few symptoms as distressing as nausea, just ask women with hyperemesis gravidarum in whom there is a dramatic deterioration of physical and social health (1). The presence of intense nausea leads patients to feel like their “body hate me”, as adeptly described in a recent qualitative analysis of this symptom in adolescents and their mothers (2). Thus, it is not surprising that nausea represents a frequent reason for referral to pediatric gastroenterologists. Much like constipation, abdominal pain, diarrhea and most other gastrointestinal symptoms, nausea may be a manifestation of a recognizable underlying intestinal or extra-intestinal disease or may be considered “functional”. Functional disorders are due to a dysregulation of the communication between brain and gut and have recently been renamed disorders of gut-brain interaction (DGBI) (3). But while the terms functional abdominal pain and functional constipation have been widely used in the past, the condition of “functional nausea” has been only recently described and endorsed. The children and adolescent Rome IV committee first proposed diagnostic criteria for this condition in 2016 (4). Since that time, studies investigating this condition have started to appear in the pediatric medical literature. This brief review summarizes what we have learnt from such reports and it is sprinkled with some of the author's personal experience in dealing with patients with severe functional nausea in his role as director of a referral center for children and adolescents with DGBI.

## Clinical Presentation

What makes functional nausea particularly challenging to address and to investigate is that there are no objective measurement tools, no established diagnostic algorithms, and no treatment guidelines. Its pathophysiology is multifactorial and likely involves autonomic, central, and gastrointestinal pathways coupled with psychological comorbidity, in the true spirit of a biopsychosocial model. The reported prevalence rates of chronic or intermittent nausea vary from 15 to 23% among school children in the United States and Central or South America (5, 6) and most of these children have functional nausea. Among children with DGBI, nausea seems to be particularly prevalent in those with pain-associated disorders and there is a 53% prevalence and in those with functional constipation (5). Nausea seems to be more common in girls and children attending private schools and there is evidence that higher nausea frequency leads to more full school days missed and being unable to do home activities (7). When stricter Rome IV criteria are used functional nausea was found to be present in 0.7% of all children (8). Nausea is also a fundamental symptom in episodic DGBI such as abdominal migraines and cyclic vomiting syndrome (CVS) and many patients with nausea fulfill criteria for postural orthostatic tachycardia syndrome (POTS). Patients with chronic nausea share many features and multi-system comorbidities with other DGBI including fatigue, headaches, dizziness, poor sleep quality, and a family history of functional disorders and the presence of nausea increases the risk that affected individuals have such other features (7). Anxiety has been reported to occur in 70% of girls with chronic nausea (9), and in a large epidemiological study in Norway the authors found that the presence of anxiety disorders was the strongest risk factor for nausea (10). In approximately half of the patients with chronic nausea, the symptom peaks in the morning. My personal experience is that morning nausea is particularly common in adolescents and often is lessened by waking up later in the morning, as often happens during the weekend. Normally, cortisol levels rise during the early morning hours and are highest at about 7 a.m. Such cortisol surge is particularly pronounced in patients with anxiety disorders and may be contributing to the onset of nausea in particularly predisposed individuals (11).

Nausea is also a cardinal symptom of gastric dysfunction with alteration in gastric electrical rhythm leading to gastroparesis or defects in fundic accommodation and triggering the nausea through abdominal vagal afferent chemo- and mechano-receptors (12). Signals transmitted to the central nervous system (CNS) may be amplified or attenuated depending on past personal experiences, environment, and psychiatric comorbidity specific to each individual. The limbic system appears to play a key role in regulating these signals. With the occurrence of nausea there are other physiologic changes such as diaphoresis, eye blinking, salivation, palpitations, and pallor. Increasing nausea perception seems to be associated with both increased sympathetic and decreased parasympathetic autonomic nervous system (ANS) modulation (13).

## Diagnosis

The relationship between nausea and gastric dysfunction has been investigated both in adults and in children. Two pediatric studies using Nuclear Medicine technology found that nausea was the only symptom linked to delayed gastric emptying in one (14) and the third most common symptom (after vomiting and abdominal pain) in the other (15). The overlap between symptoms of gastroparesis and functional dyspepsia makes studies of gastric emptying somewhat difficult to interpret. It is well-established that gastric emptying values are not predictive of symptoms severity or success of treatment (16). Yet, often patients and their families find validation of the symptom once the objective measurement of the gastric emptying of a solid meal generates an abnormal value. And when more than one gastric emptying study is done and the results diverge, invariably the abnormal result is trusted more. This is a double-edge sword because once a diagnosis of gastroparesis is made and considered the etiology of the patient's symptoms, suggestions of using treatments different from those that speed up the gastric emptying may be encountered by skepticism and lack of “buy-in”. Finally, to complicate things even more, it has been reported that nausea can also be a presentation of rapid gastric emptying, which presents with symptoms overlapping with gastroparesis (17, 18). Hence, I tend to refrain from ordering gastric emptying studies in patients with nausea unless vomiting hours after eating occurs.

There is an increasing body of evidence that a high mast cell count and eosinophilia may be found in the duodenum of patients with functional dyspepsia (19, 20). In such individuals, the immunological alterations may release mediators that increase excitability of enteric nerves resulting in hypersensitivity to a variety of stimuli such as distension and different chemicals (21, 22). It is not completely clear yet if these findings represent a primary etiology of the nausea or may be the consequence of stress and anxiety on the gastrointestinal tract. There is evidence both in the animal model and in patients with irritable bowel syndrome that long-term exposure to psychosocial stress promotes mucosal inflammation and mast cell-mediated barrier dysfunction, likely mediated by the peripheral conticotropin-releasing factor (23, 24).

The association between constipation and foregut dysfunction is well-established. Stool retention delays gastric emptying, through the so-called cologastric brake (25). Thus, often abdominal radiographs are obtained by well-meaning physicians in an attempt to uncover “occult constipation” as the etiology of the nausea. When this is done, it is common for constipation to be overdiagnosed based on the physician's subjective assessment of the colonic stool load. It should be remembered that finding a colon filled with stools is a perfectly normal finding unless one has recently undergone a colonic clean-out. Hence, a colon that has stools extending from the cecum to the rectum is a normal colon and such finding should not be relied upon to diagnose constipation in the absence of a classic clinical history or physical findings.

It has been reported that nausea is significantly associated with orthostatic intolerance during a tilt test and with decreased heart rate variability. It has been recently reported that there may be some benefit in using the head-up tilt testing to diagnose POTS or orthostatic hypotension in patients in whom there is high clinical suspicion of ANS dysregulation (26). It remains unclear though if the treatment of POTS leads to resolution of functional nausea. In addition, ANS testing is not widely available and there are often differences in testing protocols. Finally, there seems to be an association between joint hypermobility, POTS, mast cells activation syndrome (MAS) and many DGBI, including functional nausea (27, 28). I have found that the triad of functional dyspepsia, POTS and MAS represent a phenotype particularly challenging to treat and often such patients are convinced that the number of diagnoses that they carry makes them a medical mystery for which no treatment is likely to succeed. And the hopeless are unlikely to get better.

In conclusion, much like in most other DGBI, diagnostic tests in patients with chronic nausea should only be indicated in the presence of other alarm signs or features (weight loss, severe pain, bilious vomiting, etc.) (29). Upper endoscopies are particularly unhelpful with 98% reported to be normal in patient with nausea as the predominant symptom (9). Even laboratory testing rarely provides diagnostic abnormalities. A pregnancy test may be appropriate in the right age and gender, though. Performing excessive tests in patients in whom the physician believes that the symptoms is due to a DGBI is not only costly and rarely provides effective reassurance to the family, but may also lead to the diagnosis of the dreaded “incidental finding” which causes more confusion, undermines the physician diagnosis of a DGBI and leads to more testing. A recent brilliant commentary labeled such phenomenon as “unintentional symptoms intensification by doctors” and warned of the danger of diagnosing rare, yet well-defined entities, for which the diagnostic criteria are stretched to “accommodate” the patient's symptoms, such as POTS, Ehlers-Danlos syndrome, MAS, and so on (30).

## Treatment

The first randomized treatment trial of functional nausea has now been published (31). In a large study of children with functional dyspepsia or functional nausea, hypnotherapy was compared to standard medical treatment. Hypnotherapy consisted of six sessions of 50–60 min given by a qualified hypnotherapist over 3 months. The group randomized to medical treatment visited their treating pediatrician 6 times during 3 months. Pediatricians were instructed to follow a stepwise approach which used dietary changes, lifestyle modifications and several different medications given in a specific sequence. In order to correct for the patient–therapist time in the hypnotherapy group, study children additionally received six sessions of 30 min of supportive therapy. The study showed that both hypnotherapy and medical treatment were effective in reducing symptoms of chronic nausea, with hypnotherapy being more effective during the first 6 months after treatment, especially in the group of children with functional nausea. This study adds to a long list of evidence showing that cognitive behavioral treatments (CBT) and in general brain-directed therapies are very effective interventions in pain predominant pediatric DGBI (32). In fact, I am not aware of any negative study using CBT in children with pain predominant DGBI. And one could easily make the case that the “standard” medical treatment used in this study is actually an “augmented” treatment because it used four different medications, herbal supplementation and dietary recommendations.

Like in ALL pediatric functional disorders, a multidisciplinary approach which simultaneously (rather than sequentially) educates family on the brain-gut connections and the role of stress and anxiety in triggering somatic symptoms, provides reassurance and non-pharmacological interventions, emphasizes coping strategies and maintaining functioning, and involves a psychologist early in the plan is likely to provide the best outcomes (33).

Classical antiemetics such as ondansetron (a 5-HT3 antagonist) have little evidence supporting their benefit for functional nausea. STW5 is an herbal supplement, widely used in Europe, which seems to help adults and children with functional dyspepsia (34) and much like many alternative treatments is often well-accepted by parents who prefer to avoid use of medications in their children. Stimulation of the wrist acupuncture point P6 may be as effective as drugs in addressing postoperative nausea and vomiting (35). There are now several commercially available devices based on the same principle that are marketed for nausea of pregnancy or motion sickness.

Cyproheptadine is drug that has anti-serotonergic and anti-histamine effects and has shown its benefit in children with functional abdominal pain and dyspeptic symptoms including nausea (36). It is used for prophylaxis of CVS episodes and in the treatment of migraine headaches (37). It also stimulates appetite and it is particularly beneficial when the child stops eating due to early satiety and nausea and struggles in maintaining a healthy weight. When nausea is associated with gastroparesis, administration of prokinetics constitutes a rational treatment intervention. Erythromycin, a motilin receptor agonist, is the first line treatment for these cases, although its long-term use may be limited by the development of tachyphylaxis. The potential for serious adverse effects has now greatly limited the use of dopamine-2 receptor antagonists, such as metoclopramide and domperidone. Domperidone is currently available in the United States only through a Food and Drug Administration (FDA) exemption. Use of psychotropic agents such as amitriptyline, buspirone, and mirtazapine (38) which decrease visceral hyperalgesia, improve accommodation or accelerate gastric emptying may be justified in selected cases. Transdermal scopolamine patch is an effective medication for treating nausea and vomiting from chemotherapy, reducing post-operative nausea, and relieving motion sickness and has been used to treat adolescents with chronic nausea (39). The role of the neurokinin-1 receptor antagonist, aprepitant, in improving chronic rather than episodic nausea is being explored (40). Endoscopic injection of botulinum toxin in the pylorus may be beneficial regardless of the underlying etiology of the nausea (41). Cannabinoids have been studied extensively for prevention and treatment of nausea and vomiting associated with chemotherapy (42), but their chronic use may also cause dyspeptic symptoms including nausea and protracted emesis (43). Finally, there is increasing evidence that neuromodulation with implantation of a gastric pacemaker improves drug-refractory nausea, regardless of the effect of this device on gastric emptying (44). The mechanism by which such improvement is triggered is not yet fully clarified and is likely to include modulation of central, autonomic, and enteric pathways.

Given the plausible relation of chronic nausea with orthostatic intolerance and POTS, patients with nausea may also benefit from aggressive increase in hydration and salt intake, regular sleep, and exercise. The addition of low-dose fludrocortisone may improve nausea as documented by Fortunato et al. in two studies (45, 46). Obviously, when there are internalizing disorders, treatment of the anxiety and depression is also beneficial.

## Conclusion

Functional nausea is an entity that is being recognized with increasing frequency both in adults and children. Being the newest “kid on the block” among all various DGBI, the science investigating its pathophysiology has only recently started to catch up. While we eagerly await more scientific studies about the ideal testing and treatments, some facts have already been established: (1) It exists; (2) It affects the ability to enjoy age-appropriate activities; (3) Constipation may make the symptoms worse; (4) Testing is needed only in the presence of alarm features; (5) Upper endoscopy is uniformly unhelpful; (6) Gastric emptying results need to be critically interpreted; (7) Recognition and treatment of an underlying anxiety disorder is very important; (8) Most beneficial treatment is hypnotherapy.

## Author Contributions

The author confirms being the sole contributor of this work and has approved it for publication.

## Conflict of Interest

The author declares that the research was conducted in the absence of any commercial or financial relationships that could be construed as a potential conflict of interest.

## Publisher's Note

All claims expressed in this article are solely those of the authors and do not necessarily represent those of their affiliated organizations, or those of the publisher, the editors and the reviewers. Any product that may be evaluated in this article, or claim that may be made by its manufacturer, is not guaranteed or endorsed by the publisher.
